# The Allium Ureteral Stent for the Treatment of Ureteral Complications Following Renal Transplantation—A Single-Center, Single-Surgeon Series

**DOI:** 10.3390/jcm12093317

**Published:** 2023-05-06

**Authors:** Sarah Weinberger, Mandy Hubatsch, Tobias Klatte, Jörg Neymeyer, Frank Friedersdorff

**Affiliations:** 1 Department of Urology, Charité-Universitätsmedizin Berlin, Corporate Member of Freie Universität Berlin, Humboldt-Universität zu Berlin, and Berlin Institute of Health, 10117 Berlin, Germany; 2 Department of Urology, Evangelisches Krankenhaus Königin Elisabeth Herzberge, 10365 Berlin, Germany

**Keywords:** ureter, stenosis, Allium-stent, kidney transplantation

## Abstract

Ureteral complications such as urinary leak, ureteral necrosis or ureteral stenosis are common complications after renal transplantation with major short- and long-term issues, including graft impairment and graft loss. At present, there is no agreement on the optimal management of ureteral complications. The aim of the current study was to evaluate the safety and efficacy of the self-expanding, large-caliber Allium ureteral stent in patients with ureteral complications following renal transplantation. In this retrospective study, the electronic database of Charité University Hospital was screened for patients receiving the self-expandable Allium ureteral stent in the transplant ureter after kidney transplantation between January 2016 and March 2022. Descriptive statistics were used to describe the outcomes. There were six men and four women with a median age of 61 years (interquartile range, 55 to 68 years). Nine out of 10 patients had ureteric stenosis, which was diagnosed at a median of two years (interquartile range 10 months to 9 years) following renal transplantation. The median operating time was 49 min (interquartile range, 30 to 60 min). Endoscopic Allium stent placement was successful in all patients with ureteric stenosis. The median length of stay in the hospital was four days (interquartile range 2 to 7 days). Only one patient (#5) had a postoperative grade IIIb Clavien–Dindo complication. Patients had follow-ups every 3 months with ultrasound and serum creatinine. Dislocation of the Allium stent was seen in four patients; all occurred within three months. Ultimately, three patients required ureteric re-implantation, two of which had early dislocation of the stent. Six patients are managed with a permanent Allium stent. The median dwell time was 11 months (interquartile range 3 to 20 months) and maximum dwell time was 23 months. The overall success rate was 60% (6 out of 10). According to our data, the Allium stent represents a safe and minimally invasive option with a success rate of 60%. It might, therefore, represent an alternative to DJ stents, nephrostomies or immediate re-implantation. As all dislocations occurred within three months, frequent early postoperative follow-up is required.

## 1. Introduction

Ureteral complications such as urinary leak, ureteral necrosis or ureteral stenosis are common complications after renal transplantation, with a reported frequency of 2.5 to 12.5% [1,2,3]. The majority of complications arise in the early postoperative period at the site of the ureterovesical anastomosis. Ureteral complications are associated with major short- and long-term issues, including graft impairment and graft loss [4]. At present, there is no agreement on the optimal management of ureteral complications. While ureteral stents or nephrostomies are feasible minimally invasive options, success rates vary significantly. Furthermore, they are associated with major limitations such as incrustations, infections and stent obstructions [5], necessitating frequent replacement. Surgical revision of the ureterovesical anastomosis with ureteric re-implantation represents the only valid alternative.

The Allium ureteral stent (Allium Medical Solutions Ltd., Caesarea, Israel) is a self-expanding, large-caliber nitinol (nickel–titanium) stent. The stent is covered with a polymer, which prevents the ingrowth of surrounding structures and makes it non-permeable for fluids. It has shown good efficacy in some benign conditions such as post radiotherapy stricture, ureteroenteric anastomotic stricture after urinary diversion or post ureteroscopy stricture [5,6]. Additionally, due to the sealing properties (non-permeable for fluids) of the stent, there are numerous reports of successful treatment of ureteral injury, ureteral fistula or urinoma using the Allium stent [7]. In our clinical experience in ureteral injury or fistula, we see a good effect of using the Allium stent (data not published).

However, there are very limited data on the use of the Allium stent in the treatment of ureteral complications following renal transplantation. Indeed, Moskovitz et al. reported on a multicenter series of only three patients after kidney transplantation and reported a stent obstruction rate of 100% [5]. Further, no long-term data were reported in this article.

**Figure 1:**
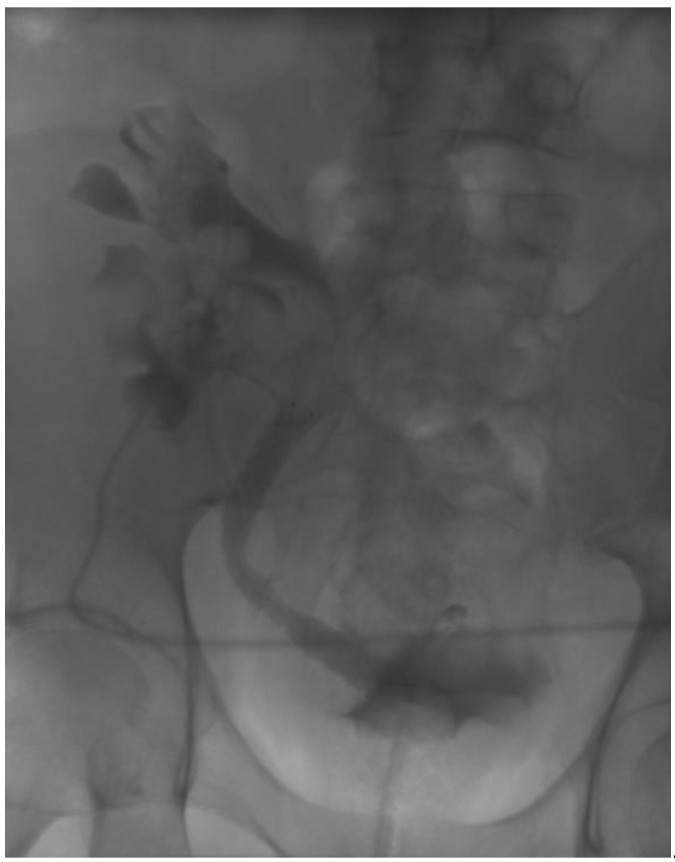
Antegrade nephrostomogram in a patient with a 120 × 100 mm Allium stent in the transplant ureter.

The aim of the current study was to evaluate the safety and efficacy of the self-expanding, large-caliber Allium ureteral stent in a larger cohort of patients with ureteral complications following renal transplantation. An example is showed in Figure 1.

## 2. Materials and Methods

The electronic database of Charité University Hospital was screened for patients receiving the self-expandable Allium ureteral stent in the transplant ureter after kidney transplantation between January 2016 and March 2022. In the present retrospective study, we identified 10 patients meeting the inclusion criteria.

The Allium URS is a fully covered, large-caliber stent. The metal self-expanding component of the stent is made of nickel–titanium alloy (nitinol). The entire stent is covered with a polymer, making it nonpermeable for fluids and preventing tissue ingrowth. The URS stent comes in two calibers (24 F/9 mm and 30 F/10 mm) and in three lengths (8 cm, 12 cm and 20 cm). It is inserted with a small diameter and spontaneously expands into and maintains a large caliber. The Allium URS comes mounted on a 10 F insertion device. The stent is inserted antegradely or retrogradely with intraoperative X-ray guidance. The URS does not shorten during or after its deployment, making its positioning accurate and stable [5,7]. All procedures were performed by a single surgeon (JN) after the patients gave informed consent to the procedure. Stent placement was performed under general anesthesia. Before the stent can be placed, a wire must be placed endoscopically in the ureter. The renal pelvic outlet and ureteral orifice into the urinary bladder must be visualized to ensure optimal stent placement. Placement of the stent usually takes a few minutes, but placement of the wire may be more difficult depending on the anatomy after renal transplantation.

Descriptive statistics were used to describe the outcomes.

## 3. Results

Patient characteristics are shown in Table 1. There were six men and four women with a median age of 61 years (interquartile range, 55 to 68 years). Nine out of 10 patients had ureteric stenosis, which was diagnosed at a median of two years (interquartile range 10 months to 9 years) following renal transplantation. Three patients had a nephrostomy before the procedure, while seven had a DJ stent in situ.

The length of the Allium stent was 12 cm in eight patients and 8 cm in two patients. The median operating time was 49 min (interquartile range 30 to 60 min). Endoscopic Allium stent placement was successful in all patients with ureteric stenosis. The median length of hospital stay was four days (interquartile range 2 to 7 days). One patient (#5, Table 1) had a postoperative grade IIIb Clavien–Dindo complication. In this case, the initially inserted stent was 8 cm in length and too short to provide adequate drainage. The stent was replaced under general anesthesia.

One patient (#9, Table 1) suffered from a complete ureteric necrosis, which was diagnosed two months following transplantation. It was not possible to place the Allium stent through antegrade or retrograde endoscopy. Conversion to open surgery was performed. There was no option of re-implantation or pyelovesicostomy due to the length of the defect, and the Allium stent was placed during open surgery. The further postoperative course was uneventful.

Patients had follow-ups every three months with ultrasound and serum creatinine until June 2022. In case of definitive surgical treatment, follow-up was finished at the date of surgery. One patient had his Allium stent removed after seven months with no functionally relevant obstruction. This patient did not require another intervention. Dislocation of the Allium stent was seen in four patients; all occurred within 3 months. The dislocated stent was removed in all four patients. Two underwent ureteral reimplantation after temporary urinary drainage, while in one patient, no further treatment was necessary. Ultimately, three patients required ureteric re-implantation, which, in our experience, can be performed as usual with the open technique. Success rate was defined as no need for additional urinary drainage with ureteral stents or nephrostomies or surgical revision of the ureterovesical anastomosis. Four patients out of six treated successfully are managed with a permanent Allium stent. In two patients, the stenosis was eliminated through dilation of the stent and no further treatment was required up till June 2022. The overall outcome is summarized in Table 2. 

## 4. Discussion

Urinary drainage is necessary to restore adequate renal function in cases of ureteral stenosis. There are two basic options to deal with ureteric stenosis. Especially in the acute situation, a stent in the ureter or a percutaneous nephrostomy can provide safe and efficient urinary drainage. Nevertheless, the necessity of frequent replacement and various side effects may have a negative impact on quality of life [5,8]. Therefore, in case of a permanent stenosis, there is the alternative of surgical revision of the ureterovesical junction to treat the stenosis permanently. However, due to poor patient condition or surgical field complexity, surgical revision may not be the preferred option [8]. According to our data, the Allium stent represents a safe and minimally invasive option with a success rate of 60%. It might, therefore, represent an alternative to DJ stents, nephrostomies or immediate re-implantation.

The large-caliber, self-expanding Allium ureteral stent ensures urinary drainage through long-term wall support of the ureter. In our population, the Allium stent provided urinary drainage for up to two years without the necessity for replacement. Early stent migration was a common problem, which is likely due to the lack of a sufficient anchoring mechanism. In line with our data, Moskovitz et al. reported stent migration in 7 out of 49 patients (14.2%) treated with Allium ureteral stents for different conditions [5]. In cases of transplant ureters, the dislocation rate may even be higher due to the shorter ureter. Other currently available stents combat migration with the presence of a proximal and distal J or pigtail, which, however, is not part of an Allium stent. There are Allium URS stents available with an intravesical anchor portion, which might reduce the migration to the proximal ureter but not towards the bladder. The extent to which the anchor could prevent the problem of stent migration needs further investigation.

As all dislocations occurred within three months, frequent early postoperative follow-up is required.

Concerning cost-effectiveness and re-intervention rates, the Allium ureteral stent competes with other ureteral stents and nephrostomies. The first-line management of transplant ureteral obstruction has shifted from open surgery to minimally invasive urinary diversion by ureteral stent [9]. There are different studies on the technique and success rate of stent placement [9,10,11]. These studies show that the minimally invasive insertion of the guidewire into the kidney, and ureter in particular, can be demanding. For example, Gerrard E.R., Jr. et al. reported a successful retrograde stent placement in 28 out of 52 cases. (53.8%). Of the 27 attempted stent exchange procedures, successful exchange was accomplished in 85.2% of cases [9]. There are only a few datasets on the dwelling time of DJ stents in transplanted patients. Halstuch et al. report a median stent dwelling time of 12.8 months [10]. There is no information given on DJ stent dislocation. However, other authors recommend a stent replacement at least once every 6 months to avoid infection or obstruction [12]. Overall, the reintervention rate for Allium stents and DJ stents does not seem to be the decisive argument. Furthermore, the duration of surgery is not determined by the stent placement itself, but by the preparatory insertion of the guidewire. Therefore, it is worth mentioning that the Allium stent is about 100 times more expensive to purchase than a DJ ureteral stent. While a DJ ureteral stent costs less than EUR 20, the price of the 120 cm Allium stent EUR 1400. Nevertheless, from our point of view, the Allium stent is an alternative that can be considered. On the one hand, only a few patients are affected (1–4% after kidney transplantation) [10], so the additional costs are hardly significant due to the small number of patients. On the other hand, there are often reasons against a permanent DJ supply or an open reoperation. Especially in patients with complaints and complications with DJ ureteral stents, an Allium stent might be a reasonable alternative.

## 5. Conclusions

More than 50% of patients with ureteric stenosis following renal transplantation benefit from an Allium ureteral stent, which appears to be an excellent alternative to conventional ureteric stents and might obviate the need for ureteric reimplantation. However, since proven alternatives exist in the form of permanent DJ supply or ureteral reimplantation, patients eligible for Allium stent placement should be selected carefully. In our clinical routine, we recommend the stent to patients who are not suitable for reimplantation or have discomfort or complications from the DJ stent.

Interestingly, two patients’ ureteral stenosis resolved after a few months with the Allium stent in place. The procedure is safe and minimally invasive; however, longer-term data are still required.

## Figures and Tables

**Table 1:** Patients’ characteristics.

*n*	Age	f/m	Type of Stent	Underlying Pathology	Diversion before Allium Stent	Interval from Renal Transplantation to Allium Stent
1	58	m	120 × 10 mm	Distal stenosis	DJ stent	3 years
2	28	f	120 × 10 mm	Distal stenosis	Nephrostomy	1 years
3	57	m	80 × 10 mm	Distal stenosis	DJ stent	15 years
4	68	f	120 × 10 mm	Proximal stenosis	DJ stent	8 months
5	78	m	80 × 10 mm	Distal stenosis	DJ stent	9 years
6	64	f	120 × 10 mm	Distal stenosis	DJ stent	8 months
7	72	f	120 × 10 mm	Distal stenosis	DJ stent	10 months
8	51	m	120 × 10 mm	Distal stenosis	Nephrostomy	12 years
9	67	m	120 × 10 mm	Ureteral necrosis	DJ stent	2 months
10	55	m	120 × 10 mm	Distal stenosis	Nephrostomy	2 years

**Table 2:** Perioperative course and long-term results.

*n*	Allium Placement	Procedure Time [min]	Date of Discharge	Length of Stay [d]	Stent Removal		Dwell Time [m]	Long-Term Result
1	22 November 2016	27	24 November 2016	2	27 February 2018	scheduled replacement	15	Allium
2	11 June 2018	38	14 June 2018	3	10 July 2018	dislocation	1	UCN 18 March 2019
3	8 November 2018	50	12 November 2018	4	14 February 2019	hydronephrosis	3	UCN 11 March 2019
4	14 February 2019	22	21 February 2019	7	-		23	Allium—Died 26 January 2021
5	23 January 2020	62	3 February 2020	11	30 January 2020	replacement	0.25	RCX 23 April 2021
6	19 June 2020	23	23 June 2020	4	September 2020	dislocation	3	NFT
7	31 July 2020	47	2 August 2020	2	3 February 2021	removal	6	NFT
8	10 September 2020	69	12 September 2020	2	5 October 2020	dislocation	1	UCN 5 May 2021
9	20 September 2021	295	28 September 2021	8	2 December 2021	dislocation	2	New Allium 13 May 2022
10	14 February 2022	54	23 February 2022	9	-		4	Allium

Data up till June 2022. Abbreviations: UCN—ureterocystoneostomy; RCX—radical cystectomy; NFT—no further treatment, the ureterostenosis was healed.

## Data Availability

The data presented in this study are available on request from the corresponding author. The data are not publicly available.
